# The patenting versus publishing dilemma

**DOI:** 10.1038/s41467-023-37243-z

**Published:** 2023-03-21

## Abstract

The process of patenting inventions may be complex. Academic researchers whose primary goal is getting their work published in scientific journals often face daunting doubts when it comes to understanding the interplay between publishing and patenting their findings. We asked Prof Frank Tietze questions from the perspective of academic researchers who wish to understand how the patenting process works and—most importantly—the relation between patenting and publishing.

**Figure:**
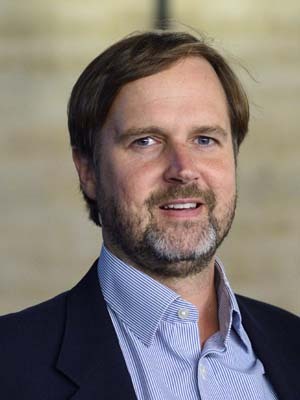
Prof Frank Tietze

*Frank Tietze* is Professor of Innovation Engineering at the University of Cambridge. Research at his Innovation and IP Management (IIPM) laboratory focuses on the role of Intellectual Property (IP) in collaborative innovation processes and systems for emerging and sustainable technologies. However, as he is not an IP lawyer, this Q&A should not be regarded as legal advice.

**How do I determine if an invention is patentable?**

It is important to understand that patent systems are not identical globally (in fact, there is nothing such as a global patent system) and countries have slightly different rules. However, patent laws in different countries have been gradually harmonised in the last few decades, where at their heart lie three fundamental and general criteria that patent offices typically use to determine patentability.

The first criterion is ‘novelty’, which refers to being ‘novel to the world’. This means that any invention that you file for patent protection, must not have been made public anywhere before filing the application with a patent office.

Making public refers not just to being published in academic journals, but also refers to all kinds of other ways of publishing, such as conference posters, newspapers, industry magazines, exhibitions, even personal online blogs and videos, literally anywhere where the public would have access to it. It really needs to be said that you got to be very careful with this criterion as even a verbally made statement (if this could have been recorded), such as a presentation at a conference can create problems. Novelty also extents to publications in any language, which nowadays patent examiners can find using machine translation services.

Being able to demonstrate novelty is ultimately important for the patent office when examining if your application should be granted a patent, but potentially also at a later stage. Assume that a patent examiner does not identify any novelty problems; you might end up with a granted patent. However, if any public disclosure exists from before the patent application date, this can be used by anyone to invalidate the patent. You might say, who would do this? Here is an example. Imagine that your patented invention becomes commercially valuable, say because it gets sold to a large company or becomes the core of a successful start-up. At some point a competitor might very well have an interest to challenge it. Such party can then use public disclosures that were made before the patent application date and argue that ‘prior art’ existed wherefore the patent should not have been granted in the first place. Remember, today the internet does not forget! Obviously, invalidating a patent can be an extremely costly and lengthy endeavour as it is likely to result in a court case, but is not impossible.

The second criterion refers to ‘inventiveness’ (non-obviousness). Any invention for which an applicant seeks patent protection will need to be inventive. While some inventions might be considered breakthroughs, i.e., with a high inventive step, the majority of patents tends to represent incremental improvements. Hence, for the patent examiner, the question usually is what a sufficient, minimal inventive step would be to warrant a patent being granted. There is no exact measure for this, but the case that is often made is the reference to the ‘person skilled in the arts’. The argument here is that if a person, who is an expert in the field of the invention, would be ‘surprised’ by the invention and not consider it to be trivial, this indicates a sufficient inventive step. It is, however, worth mentioning that even today low-tech inventions can still be patentable if they can demonstrate a sufficient, non-obvious inventive step. In fact, I have seen quite several successful examples of those, such as an aerofoil wing, a small piece of wind-channel tested and carefully designed plastic that is attached to supermarket fridges substantially reducing their energy consumption.

Third, an invention for which the applicant seeks patent protection needs to be ‘industrially applicable’. Essentially, this criterion ensures that patents are only granted for technical inventions. Hence, it is used to exclude non-technical inventions from being patented, such as new songs and poems, which tend to be subject to other means of protection, such as copyright. Related to this it is worth noting that certain subject matters are excluded from patenting, such as scientific principles, laws of nature, naturally occurring chemical compounds, business models or software algorithms.

However, given the enormous importance of software in today’s economy it seems important to clarify that this does not mean that software per se is not patentable. If software can be shown to have a further technical effect (e.g., ‘embedded software’), it might very well be patentable, at least in some important jurisdictions, such as Europe. An example is energy-management software that reduces energy consumption of elective vehicles or data centres.

Finally, it is worth mentioning that universities’ Technology Transfer Offices (TTO) and companies’ IP departments are usually fairly informed about these general patentability criteria and can provide advice, if not a first assessment of whether an invention can possibly be patented. If in doubt, patent attorneys are the next port of call, however, one should not forget that they make a living by drafting and submitting patent applications. It would be best to first seek an independent opinion. Unfortunately, there are not many independent service providers that have a more neutral view. The few that could be mentioned here besides TTOs are Patent Libraries (PATLIB) centres and IP helpdesks, such as the one operated by the European Union.

**Will submitting a patent application prevent me from publishing my research?**

Submitting a patent application should not prevent you from publishing the related research. It is important to understand that when filing a patent application with a patent office the application only gets published 18 months afterwards. In other words, the patent application will still be kept secret for that period. If you consider submitting a paper to an academic journal after the patent application has been published (or even within the 18 months), you can even consider referencing the patent application. I am not aware of publishers who have rejected papers because they reference corresponding patents from the author(s). In most cases, the techno-legal language used in patent documents tends to be anyway quite different from the language and wording we use in academic papers. Hence, most likely, text that is used in patent documents will be altered for academic publications, therefore, there should not be any copyright related problems.

The other way around is more likely to present a problem, possibly a serious problem. If you publish a paper first and then want to file a patent application you can encounter problems with the ‘novelty’ criterion, which can ultimately lead to the patent application being rejected. Novelty is a rather universal criterion that almost all patent offices apply and only some very few countries allow patents to be granted after the invention has been publicly disclosed before the application date, but only if one can prove that it is your own work and only for a limited time after the public disclosure. For instance, the US allows a so-called grace period as well as exhibiting exceptions.

In any case, I would always advise being very careful with publishing if you have made an invention that can be considered patentable. I suggest contacting the respective department in your organisation, for instance, the TTO if you work at a university as early as possible. Your TTO would then ask you to complete an ‘invention disclosure’ form. This allows them to open a case so they can discuss possible publishing scenarios with you. However, filing an invention disclosure form does not mean you can move on and publish your paper without having to worry about being able to demonstrate novelty to the patent office.

**Can a grant application be considered a public disclosure preventing me from obtaining a patent? What about a scientific presentation at a conference?**

Grant application processes typically tend to be confidential, so should not count as public disclosure. One might though be careful about the documents that funding bodies publish ones they have decided to fund a project. Typically, at least, they publish short project descriptions on their websites. One would want to be careful not to disclose anything relevant to the invention in such project description. Researchers who received grant funding might want to report any patent application that results from a grant back to the funding body.

When presenting at a conference, I would say you should be careful about anything that could potentially be deemed inventive and relevant for a patent application. There are different conference formats. Some conferences do not publish proceedings and others do. One should be particularly careful with regards to the latter. In order to be on the safe side, I would advise to speak to the relevant department in your organisation, such as the TTO not only a day or two before the conference. TTO colleagues can then decide whether to file a patent application (or a provisional application, e.g., in the US) before the conference. There have been cases in which patent applications were prepared in a rush to be filed just before the conference, even on the same day, which is far from ideal as this can negatively impact the quality of those patents.

If an issue arises late and one has already submitted a manuscript to a conference, one can ask the organisers to exclude the paper from the proceedings to avoid public disclosure or withdraw it from the conference. For some conferences, participants actually submit (extended) abstracts. In that case, one could write the abstract in such a way as to not disclose inventive elements. Finally, one needs to be careful with regards to the presentation during the conference. Even content that is disclosed verbally or shown on presentation slides can be considered a public disclosure.

**Is there a difference between a co-author on a publication and an inventor on a patent? If so, how are inventors determined?**

There are some similarities, but also some important differences. The authors of academic publications are typically those who contributed in some capacity to the research that a publication reports upon. The copyright is the specific IP right that is relevant for academic publications. When writing a publication, the authors automatically create/obtain the copyright for the manuscript. When authors submit manuscripts to publishing houses, at least in the traditional (i.e., non-open access) model, publishers tend to ask the authors to reassign their ownership of the copyright to them.

When it comes to patents, the inventors are those who contributed to developing an invention that is described in the corresponding patent document. The inventors themselves might want to draft the patent application or (as is often the case) describe the invention to a patent attorney, who then drafts the patent document (i.e., being trained to write the patent in a specific techno-legal jargon). Hence, while inventors might actually not be those who ultimately have written the patent document, it is even more important to understand that inventors might not be those who end up owning the invention described in the patent document. Whether inventors own their inventions is typically determined by their employment contracts. Nowadays, employment contracts, whether for large firms, start-ups, commercial R&D institutes, but also universities, tend to include clauses that govern the ownership of the IP developed by employees. In fact, in most cases, employees assign their IP to their employer. The employer is then listed as applicant (or assignee in the US) on the patent document. It is also worth mentioning that the sequence in which inventors are listed on patent documents not necessarily reflects the contributions of the inventors as it tends to be the case for academic publications, at least in some disciplines.

In summary, patent documents typically list three parties: the inventor(s) as those who developed the invention; one or more applicants/assignees as the patent owner; some patent documents also contain information about patent attorneys. In contrast to the inventors who must be natural persons, patent owners can be legal entities, such as universities, research institutes, and companies, but also not-for-profit organisations, such as charities. There are current discussions about the role of artificial intelligence algorithms as inventors and/or applicants. I am still waiting to hear from legal colleagues how this will be ultimately decided.

**I have published a patent. Can I use the same figures or schematics for the paper I am writing?**

The answer to this question seems to lie in another question, i.e., who owns the copyright of a patent document. The legal situation appears to differ across countries. For instance, in the UK, applicants own the copyright of patent documents, while it is the opposite for applications submitted to the European Patent Office (EPO). Whatever the case, it seems very unlikely to me that a patent office would take legal action against inventors for reproducing drawings or figures from corresponding patent documents. I do not think I ever heard about such a case. In this regard, we have to remember the original purpose of patent systems. They have been installed to facilitate knowledge sharing by means of incentivizing the disclosure of knowledge in patent documents so that others can build upon it, at least after patents become inactive (e.g., due to non-payment of renewal fees) or expiry after their maximum term, which is typically 20 years. Originally the word ‘patent’ comes from the Latin ‘patere’ meaning to lay open.

**Would you consider it useful to file a patent? What are the benefits, and what are the risks?**

Patents have a role to play in our common pursuit of scientific and technological progress more generally, not the least because they encourage public disclosure of technical and inventive knowledge which otherwise might stay secret. Only if knowledge is being disclosed, other can build on it (if patented, though maybe with a delay). In fact, over the years I came to understand that patent databases can be considered the world’s oldest and largest open-source repositories for technical knowledge.

It is important to understand that patents essentially do nothing more than allocating ownership rights to an invention. In classic economic theory the clear allocation of ownership is an important determinant for functioning markets. Hence, filing a patent application (and ultimately its grant) allocates the rights of ownership for the invention, thereby putting the owner in the position to make decisions about what happens to the invention. One of the biggest misconceptions I have heard many times is that patents as such prevent progress and prevent others from using inventions. This is wrong as patents only put owners in a position to make decisions about what happens to their inventions. In other words, patents allow their owners to control the usage of their inventions. The allocation of ownership rights puts owners in a position to make choices and be selective about the use of their inventions. Importantly, this does not mean that a patent prevents other from using an invention, but only puts the inventor in a position to do so, if she wishes so.

For instance, owners can choose to let others use their invention for free. There are various examples of so-called patent pledges. In 2014, a well-known US electric vehicle company publicly announced that others can use their patents for free. It is important though to understand that this does not mean the company is giving up the ownership of its patents, but essentially provides anyone who wishes to use their inventions with a free license. Many such pledges have been made over the years. While some pledges come with strings attached, this should just be seen as an example that patents put their owners in a position to choose what they want to do with their inventions. In a way, a pledge is a construct similar to what we know as open-source software. Open-source software means that the creators use royalty-free licensing to allow others to use their code while the creators still maintain the ownership of the software, even though ownership is typically not allocated by patents but by copyright. Another option that owners of patented inventions have (which is probably perceived to be the ‘classical’ option) is that they can either prevent others from using their invention or charge them a royalty fee through a contractual agreement typically known as a license. This option is often chosen for competitors, but patents can also be used by their owners to prevent other market actors from using their inventions, such as defence firms or certain companies that do not share their same values, e.g., with regards to sustainability.

An analogy that I sometimes use in my lectures is the ‘front door of a house’. Imagine your house has not got an entrance door. Without that door, anyone can come in, sit at your kitchen table and grab a drink from your fridge, maybe even sleep in your bed. Without such a door, you might find it difficult to control the use of your house. Would you want that? Maybe not. Hence, installing a door puts the resident in a position to make decisions about whom to let into the house. Even with a door you can have a liberal open-door policy and invite friends to come over and help themselves to a drink. Some of them may even decide to stay for dinner and overnight. But having a door also allows you to be more restrictive, for instance, inviting only close family. Hence, the door (i.e., a patent) enables the resident (owner) to be in a position to make decisions. Without it, it would be difficult to govern who will be around the house, i.e., who will be using an invention.

On top of all of this, there are other considerations of course. Patents obviously can be costly. These costs can usually be split between costs for obtaining, maintaining and enforcing the patent. Drafting patents is often done by patent attorneys, who might be more or less expensive, depending also on the input that inventors provide themselves. Particularly if one considers an invention to potentially be commercially valuable, investing more at this stage should be considered as it might result in a better crafted (e.g., broader, more robust) patent. After a patent application has been submitted to the patent office (fees are typically not that high) additional costs tend to arise from the need to interact with the patent examiner during the examination process, which usually also happens via a patent attorney, who charges for that. Depending on chosen routes for filing a patent application, after some time, the applicant needs to decide in which countries to seek patent protection. Translating highly techno-legal documents into different languages can become quite costly. And, finally, you need to pay renewal fees every (few) years to keep patents alive.

For an invention to be protected in a few countries with patents kept for 8–10 years costs might easily sum up about £/€/$50k. Clearly, this is a lot of money. However, in comparison, this seems to be roughly one annual salary of a junior R&D engineer, plus the costs spread out over some years. If a patent protects an invention that forms the basis of a start-up or successful product line that investment might be worth considering. Finally, one should not forget that there might be costs potentially associated with enforcing a patent, i.e., in the worst case for a patent lawsuit. Unfortunately, this is where it can get very costly. However, some companies offer patent insurances and litigation finance. This is obviously a major risk and potential downside. However, again, if a patent protects the backbone of a start-up the immediate upside of this patent (e.g., to convince investors) might be much higher than the eventual downsides.

**How do I determine in which country I should submit a patent application? Is the process country-dependent, and will my patent be valid worldwide?**

First and foremost, it needs to be said clearly: there is no ‘world patent’. The relevant concept in this context is what is known as ‘patent family’. Patents are jurisdictional, i.e., national patent offices can only grant patents for their country. In Europe, since the European Patent Convention in 1973, we have a European patent system that is operated by the EPO. This system is currently being complemented (if not ultimately superseded) by the Unitary Patent System, which should make it even easier to obtain ‘truly’ European patents and enforce them across Europe.

What is a patent family? While, strictly speaking there are different definitions, a patent family is often considered to be the set of patents that are granted (or pending applications currently under examination) in different countries that eventually protect the same invention. After the first (also called priority) patent application in one country, applicants typically have 12 months to decide in which countries they want to seek patent protection. Patent applications will then need to be filed and eventually translated into the national languages of those patent offices.

Why would one seek patent protection in different countries? While there is a multitude of reasons to be considered on a case-by-case basis, I would say there are two more general reasons that should be considered. The first one is the choice of countries in which applicants want to control who they want to allow producing/manufacturing an invention, e.g., embedded in a product. The second reason is to decide the countries in which one wants to control the distribution or sale/usage of an invention. In other words, one wants to seek patent protection in countries where there are reasonably sized markets.

While the second reason might be more obvious, let me try to illustrate the first reason with an example. One of the case studies we covered in our research is an award-winning company that distributes highly nutritional products to beneficiaries in low- and middle-income countries, particularly in Africa. While the company did not seek patent protection across African countries (mostly because those countries have weak patent regimes, therefore it is difficult to enforce patents there), it decided to file patents in Global North countries in which multinational consumer goods companies have manufacturing sites. Thereby, they were able to prevent those companies from manufacturing and exporting products for a lower price into African counties, which would have undermined the company’s social-impact-focused business model as part of which the company aims to create higher-skilled and higher-paid jobs using local resources in Africa.

*This interview was conducted by Nature Communications editor Dr. Silvia Milana*.

